# Artificial intelligence in allergology: A Position Paper of the German Society of Allergology and Clinical Immunology (DGAKI), German Academy of Allergy, Clinical Immunology and Environmental Medicine (AeDA) and German Society of Pediatric Allergology and Environmental Medicine (GPA): Part 1

**DOI:** 10.5414/ALX02643E

**Published:** 2026-10-12

**Authors:** Oliver Pfaar, Stephan Traidl, Friederike Bärhold, Sven Becker, Christoph Bergmann, Jeroen Buters, Stephanie Dramburg, Wojciech Francuzik, Michael Gerstlauer, Eckard Hamelmann, Camilo José Hernandez Toro, Ludger Klimek, Lars Lange, Paolo M. Matricardi, Laura Pfeffer, Mariel Suarez-Suarez, Philipp Utz, Martin Wagenmann, Margitta Worm, Mehrak Yoosefi, Sonja Mathes, Sebastian Seurig

**Affiliations:** 1 Department of Otorhinolaryngology, Head and Neck Surgery, Section of Rhinology and Allergy, University Hospital Marburg, Philipps-Universität Marburg, Marburg,; 2 Department of Dermatology and Allergy, Medizinische Hochschule Hannover, Hanover,; 3 Center for Rhinology and Allergology, Wiesbaden,; 4 Department of Otorhinolaryngology, Head and Neck Surgery, University of Tübingen,; 5 Hals-Nasen-Ohrenheilkunde, interdisziplinäre Facharztklinik, Düsseldorf,; 6 Center of Allergy & Environment (ZAUM), Member of the German Center for Lung Research (DZL), Technical University and Helmholtz Center, Munich,; 7 Department of Pediatric Respiratory Care, Immunology and Intensive Care Medicine, Charité – Universitätsmedizin Berlin, Corporate Member of Freie Universität Berlin and Humboldt-Universität zu Berlin, Berlin,; 8 Allergology and Immunology, Department of Dermatology, Venereology and Allergology, Charité-Universitätsmedizin, Berlin,; 9 Pediatric Pulmonology and Allergology, University Hospital Augsburg, Augsburg,; 10 Children‘s Center Bethel, University Hospital OWL, University Bielefeld, Bielefeld,; 11 Institute of Biometry and Clinical Epidemiology, Charité – Universitätsmedizin Berlin, Berlin,; 12 Department of Pediatrics, GFO-Kliniken Bonn, St. Marien-Hospital, Bonn,; 13 Institute of Allergology, Charité – Universitätsmedizin Berlin, Corporate Member of Freie Universität Berlin and Humboldt-Universität zu Berlin,; 14 Fraunhofer Institute for Translational Medicine and Pharmacology ITMP, Allergology and Immunology, Berlin,; 15 Pediatric Dermatology and Allergology, Kinder- und Jugendkrankenhaus AUF DER BULT, Hannover,; 16 Kinderarztpraxis Hirschberg, Hirschberg,; 17 Department of Otorhinolaryngology, Düsseldorf University Hospital, Düsseldorf,; 18 Kinderarztpraxis Dr. med. Mehrak Yoosefi, Berlin,; 19 Department of Dermatology and Allergology, TUM Clinic Rechts der Isar, School of Medicine and Health, Technical University of Munich, Munich, and; 20 Department of Respiratory Medicine, Allergology and Sleep Medicine, General Hospital Nuremberg, Campus North, Paracelsus Medical University, Nuremberg, Germany; * Contributed equally as first authors.; ** Contributed equally as last authors.

**Keywords:** artificial intelligence, machine learning, allergology, immunology, CDSS, environmental analytics, patient education

## Abstract

Background: Artificial intelligence (AI) is rapidly advancing in medicine, yet its translation into clinical and research practice in allergology remains limited. Given the heterogeneity of allergic diseases and the complexity of integrating clinical, environmental, and molecular data, a structured assessment is required. This first part of a joint position paper, developed by German allergology societies and associations under the leadership of the DGAKI, aims to establish a conceptual and clinical foundation for AI in allergology. Materials and methods: The Position Paper is based on a targeted narrative literature review and structured expert consensus. Development was informed by AGREE II principles. Part I comprises Chapters 1 – 4, covering introduction, methodology, AI taxonomy, and selected early application domains. Results: AI is conceptualized as an augmentative tool supporting clinical reasoning and individualized decision-making. The paper distinguishes classical statistical approaches from machine learning, deep learning, vision models, and large language models, emphasizing their complementary roles. Key applications include environmental analytics (e.g., automated pollen monitoring and forecasting), image-based classification systems, clinical decision support for diagnostics and therapy, and emerging patient-facing tools. Across these domains, AI may improve diagnostic precision and enable integration of heterogeneous data. Limitations include restricted generalizability, bias, limited explainability, and regulatory and data protection challenges. Conclusion: The role of AI in allergology is defined, its methodological basis is outlined, and initial application domains are highlighted. Together, these elements provide a framework for evidence-based and patient-oriented integration, requiring human oversight, robust scientific validation, and regulatory alignment.

## 1. Introduction 

Artificial intelligence (AI), particularly methods of machine learning (ML) and data-driven modeling, is becoming increasingly important in biomedical research and clinical care. In allergology, a medical discipline characterized by pronounced clinical heterogeneity, complex immunological networks, and a wide range of relevant influencing factors, AI offers the potential to substantially extend existing concepts of diagnostics, phenotyping, patient information, patient consent, and therapeutic decision-making. International professional societies and study groups have emphasized the need to integrate innovative digital technologies into care of atopic diseases in a structured, evidence-based, and responsible manner [1, 2, 3]. 

A growing body of evidence already suggests the feasibility of selected AI applications in allergology. ML-based approaches have been applied to the phenotyping and endotyping of asthma, allergic rhinitis, and atopic dermatitis, frequently using clustering algorithms and multivariate modelling strategies [4, 5, 6, 7]. Additional fields of application include AI-supported analysis of molecular allergy diagnostics, including diagnosis and allergen immunotherapy selection for seasonal allergic rhinitis, component-resolved diagnostics, with the aim of improving the clinical interpretation of complex sensitization patterns [5, 8, 9] and estimating the risk of severe systemic reactions, in Hymenoptera venom allergy [8] and β-lactam allergy [10, 11, 12] as well as the likelihood of reaction in oral food challenges [13, 14, 15]. Furthermore, predictive models for treatment response to allergen-specific immunotherapy [16] as well as for the selection of targeted biological therapies [17] have been described. 

The potential added value of AI in allergological care can be seen particularly in the integration of heterogeneous data sources, improved diagnostic precision, and support for individualized therapeutic decision-making [4, 5, 6, 18]. AI-based systems can help to capture interindividual variability more effectively and may contribute to the standardization of complex clinical assessments [4, 5, 6, 17]. Importantly, AI is not intended to replace medical expertise but rather to augment clinical reasoning and support evidence-based decision-making, as emphasized in recent work on clinical AI [19, 20]. 

Despite these promising developments, relevant limitations remain. These include limited generalizability of many models, potential bias resulting from non-representative datasets, a lack of data transparency, explainability (“black-box” models), and unresolved ethical, legal, and regulatory issues. Standardized validation, robust clinical evidence, and clear governance structures are therefore essential for responsible implementation [4, 8, 21]. 

Furthermore, there is a risk that physicians who are inexperienced in allergology will invest less time and care in their own training and continuing education in the field of allergology, as they can obtain medical information for self-education more quickly and easily via freely accessible AI. This information may be incorrect or at least incomplete, leading to potentially dangerous misjudgments, especially when patients use AI to make self-diagnoses and treatment decisions [22]. 

The aim of this position paper is to assess the current state of AI in allergology, define clinically relevant fields of application, critically appraise opportunities and limitations, and formulate consensus-based recommendations for the evidence-based, safe, and ethically responsible implementation of AI in research and clinical practice. 

### General areas of application 

Beyond medicine, AI technologies are widely used in fields such as image and speech recognition, natural language processing, autonomous systems, financial risk modelling, and environmental analysis. In these domains, ML and deep learning (DL) approaches have demonstrated the ability to extract relevant patterns from high-dimensional data, automate complex analytical processes, and achieve predictive performance exceeding that of traditional methods of data analysis [23]. 

In medicine, applications range from image- and signal-based diagnostics to risk prediction, clinical decision support, workflow optimization, and personalized treatment planning. AI-driven models have shown promising performance in integrating multimodal data sources, such as clinical records, imaging, laboratory results, and molecular profiles, with the aim of improving diagnostic accuracy and patient stratification. Rather than replacing clinicians, contemporary medical AI is increasingly framed as technology augmenting healthcare, designed to support clinical reasoning, reduce variability in complex assessments, and assist in individualized therapeutic decision-making. Narrative and systematic reviews in medical AI consistently underline that clinical benefit depends not only on algorithmic performance but also on careful validation, interpretability, and seamless integration into real-world clinical workflows [24]. 

### Conclusion 1 

Rather than replacing clinicians, medical AI is increasingly conceptualized as an augmentative tool that supports clinical reasoning, reduces variability in complex assessments, and facilitates individualized treatment decisions. Evidence from narrative and systematic reviews indicates that clinical benefit depends not only on algorithmic performance, but also on robust validation, interpretability, and effective integration into clinical workflows. 

## 2. Materials and methods 

This Position Paper is based on a non-systematic narrative review of the literature combined with structured expert input. A targeted literature search was conducted from November 2025 to January 2026 in PubMed to identify relevant publications on the application of AI in allergology. After inclusion of additional relevant literature during drafting the manuscript, the review of the literature covered the period from November 2025 to March 2026, providing expert consensus on the content of each chapter of this Position Paper. All chapters were then reviewed and discussed by all co-authors; in cases of dissent, iterative discussion and feedback was applied to achieve consented texts, with agreement of at least 70% of authors necessary for each debated statement. Used combinations of keywords included artificial intelligence, machine learning, allergy, allergic disease, atopic disease, clinical decision support, digital health, and patient engagement. 

This review focuses on peer-reviewed articles addressing clinically relevant applications of AI in allergology, such as diagnostic support, risk prediction and phenotyping, treatment optimization, automation of clinical or laboratory procedures, patient-facing digital tools, and ethical, legal, and societal considerations. In addition to original research articles, gray literature, legal texts, narrative reviews, commentaries, and perspective papers were included when they provided conceptual insights, highlighted unmet needs, or addressed patient-centered and implementation-related aspects. Further relevant publications were identified through manual screening of reference lists of key articles. 

Given the rapidly evolving nature of AI technologies and their translation into clinical practice, this review was designed as a focused, narrative synthesis rather than a systematic review. The objective was to identify, appraise, and integrate representative, influential, and clinically relevant evidence addressing key questions related to the application of AI in allergology patients. Evidence selection was guided by relevance to clinical decision-making, methodological rigor, and impact on practice. This approach was chosen to inform practical guidance, while explicitly acknowledging the strengths and limitations inherent to a non-systematic evidence synthesis. 

The development of this Position Paper was informed by the AGREE II reporting framework to enhance methodological transparency and reporting quality. Although the present manuscript does not represent a formal clinical practice guideline with graded recommendations, several core domains of the AGREE framework were considered during manuscript preparation. These include definition of scope and objectives, transparent description of the development group, expert consensus procedures, and disclosure of competing interests. The AGREE principles were applied pragmatically to improve clarity, transparency, and reproducibility of the consensus process [25]. 

### Conclusion 2 

This Position Paper has been developed with reference to the AGREE II framework aimed to enhance transparency and reporting quality. Although not a formal guideline, key domains, such as scope, authorship, consensus methods, and conflict of interest disclosure, were addressed and AGREE principles were applied pragmatically to improve clarity and reproducibility. 

## 3. AI taxonomy 

### 3.1. Distinction between AI and methods of classical statistics 

Classical statistical methods and AI methods may appear as completely separate entities. In practice, both aim to leverage data to solve particular tasks. Clinicians trained in epidemiology and biostatistics might be more familiar with classical statistical methods. We aim to provide a brief introduction to AI, highlighting overlaps with classical statistics, while also drawing clear distinctions between AI methods and classical statistical and data analysis methods.

- Classical statistics: In medical research and clinical epidemiology, classical statistical methods were and still are widely used. Classical statistical methods cover linear and logistic regression, survival analysis, and generalized linear models. These methods are usually, but not exclusively, built around hypothesis-driven analysis, where a suitable model structure is predefined based on expert-guided assumptions made about the underlying data. The main goals are usually interpretability and making inferences about population parameters to understand the underlying mechanisms of health and disease or evaluate treatment effects, among others. Thus, these approaches aim to describe the relationships between variables present in the data, but do not optimize for the predictive performance of the models and rely heavily on expert knowledge for their specification and interpretation.
- Artificial intelligence: AI is a broad term that describes computer systems that leverage large corpora of training data to synthesize pattern-recognition algorithms and allow for knowledge transfer. In recent years, this field of knowledge, together with related subtopics, such as ML, DL, and more recently, large language models (LLMs), has gained popularity across a great variety of disciplines including medical and life sciences.

Together, these approaches are complementary: classical statistics offers interpretability and potential causal insights, while AI, in particular ML methods, provide powerful tools for prediction and pattern recognition of large data sets and complex data. 

### Conclusion 3 

Classical statistical and AI methods may seem distinct, but both aim to leverage data to address specific tasks. Therefore, it is fundamental to understand both distinctions and overlap between both analytical approaches. 

### 3.2. The different forms of AI 

**Machine learning**

As opposed to most of the classical statistical methods mentioned before, ML encompasses data-driven methods that focus on optimizing predictive performance or on discovering hidden structures in the data rather than on inference and interpretability [26]. The main difference between these approaches and traditional statistics is not purely mathematical, but rather related to the workflow and objectives. Instead of using expert knowledge during model selection and to determine the assumptions made about the data-generating mechanism, ML algorithms – such as random forests, gradient boosting, or neural networks – learn patterns directly from data by iteratively optimizing model parameters. As a result, these methods can represent complex relationships and interactions within the data without explicit model specifications [26]. Thus, ML places greater focus on evaluating how well a model performs on new, unseen data, than on the interpretability of the model itself. 

**Deep learning**

A branch of ML known as DL uses neural networks with multiple layers that automatically learn hierarchical representations of data. These methods can be applied to most tasks where ML methods are suitable, and are particularly powerful dealing with high-dimensional unstructured data [27], such as medical images, ECG waveforms, and free-text clinical notes. DL models often operate as black boxes, making interpretability more challenging given their complexity [28]. 

**Vision models**

Vision models are AI systems designed for the analysis and interpretation of visual data, such as images or video frames. In medical applications, including allergy and immunology, these models are predominantly based on DL architectures, most notably convolutional neural networks (CNNs) and, more recently, vision transformers (ViTs). Their primary objective is to extract reproducible and quantitatively interpretable features from pixel-level data. 

**Convolutional neural networks**

CNNs represent the most established vision architecture in medicine. They learn hierarchical image features, ranging from low-level patterns (edges, contrasts) to complex disease-associated structures. In allergology, CNN-based approaches have demonstrated high performance in image-driven tasks, particularly in dermatology. CNN-based systems have been developed to support the assessment of urticaria activity, conjunctival allergen provocation tests, and skin prick test reactions, with evidence suggesting reduced interobserver variability compared with manual evaluation [29, 30, 31, 32]. 

**Vision transformers**

ViTs apply attention-based mechanisms, which enable context-dependent weighting of relevant information by the model itself to image patches and allow modeling of global spatial relationships [33]. While their use in allergology is still largely exploratory, they are increasingly relevant for multimodal systems that integrate visual data with clinical text. Their broader adoption is currently constrained by substantial data requirements and computational complexity [29]. Beyond clinical imaging, vision models are also applied in environmental allergy research. DL-based pollen classification from microscopy images, through vision models, has demonstrated high accuracy but remains challenged by particle overlap, limited depth information, and morphological similarity between taxa, emphasizing the importance of careful dataset curation and external validation [34, 35]. 

**Large language models and transformers**

LLMs and the related reasoning models are recent developments in DL. These have gained popularity because of their use in chatbots, such as ChatGPT, Claude, Google Gemini and the European LLM Mistral. These models are trained on incrementally large collections of text in order to recognize probabilistic associations between words and generate text, thus collectively being considered types of generative AI. Their defining feature is the use of self-attention mechanisms, which allow contextual dependencies across long text sequences to be modeled efficiently. In medicine, LLMs enable analysis of unstructured data such as clinical documentation, guidelines, and patient-generated queries. 

In allergology, LLMs have been evaluated for knowledge retrieval, clinical support, and patient education. Several systematic assessments indicate that responses to allergology-related questions are often coherent and largely factually correct. However, consistent limitations in reliability and transparency have been reported. An EAACI-affiliated evaluation of ChatGPT-4 responses on allergen immunotherapy rated overall information quality as fair, while reliability scores were insufficient due to absent authorship, references, and disclosure [36]. Importantly, LLMs generate text based on probabilistic likelihood rather than explicit fact verification. 

A systematic analysis of 120 interdisciplinary allergology questions identified not only minor inaccuracies but also a small number of clinically relevant errors with potential safety implications, particularly in pediatric and food allergy contexts [22]. These findings underscore that current LLMs cannot – or should not – be considered autonomous clinical decision tools, although they could be useful in a blended human–AI approach (human-in-the-loop and human-on-the-loop) [37]. 

Ongoing research therefore focuses on domain-specific adaptation strategies, including finetuning and retrieval-augmented generation, which explicitly ground model outputs in curated medical sources such as guidelines and peer-reviewed literature [38]. Within an AI typology framework, LLMs should be regarded as powerful language-processing systems with supportive roles in allergy care, requiring continuous expert oversight. 

### Conclusion 4 

ML algorithms, such as random forests, gradient boosting, and neural networks, derive patterns from data through iterative parameter optimization. Consequently, emphasis is primarily placed on generalization performance to unseen data rather than on model interpretability. 

## 4. Applications for AI in allergology 

### 4.1. Environmental analytics 

**4.1.1. Pollen analysis**

Pollen and allergies

AI has the potential to facilitate the integration of exposomics into allergy care. Exploring possibilities to do so via pollen monitoring seems promising as a use case detailing the high granularity of data in exposomics. As Europe is characterized by a highly heterogeneous distribution of allergenic plant species, resulting in substantial regional differences in pollen exposure [39, 40], the allergenic burden experienced by sensitized individuals strongly depends on geographic location. 

In Northern and Central Europe, the predominant aeroallergens originate from pollen of the Betulaceae family, particularly birch (*Betula*), hazel (*Corylus*), and alder (*Alnus*) [41, 42]. In contrast, in Mediterranean regions, pollen from the Oleaceae family, including olive (*Olea*), ash (*Fraxinus*), and privet (*Ligustrum*), represents a major source of allergenic exposure [43, 44]. Across Europe, however, among other important allergens such as weed pollen, including mugwort (*Artemisia*) and ragweed (*Ambrosia*), pollen of the Poaceae (grass) family remains the most common aeroallergen and a leading cause of seasonal allergic rhinitis [45, 46, 47]. 

Pollen monitoring in the world

Individuals sensitized to pollen rely on information about local pollen concentrations to better manage symptoms and adapt daily activities to environmental exposure. To monitor airborne particles such as pollen, and frequently also allergenic fungal spores including *Cladosporium* and *Alternaria* spp., aerobiological monitoring stations have been established worldwide (Figure 1a). 

Traditionally, pollen monitoring has relied on manual sampling devices, particularly Hirst-type traps, which require microscopic identification by trained experts and typically provide results with delays of several days. These approaches are labor-intensive and subject to methodological limitations [48, 49, 50, 51]. 

In recent years, automated monitoring systems integrating AI-based image recognition have emerged, and it is now possible to avoid some of the mistakes of the manual traps [52, 53, 54]. With automatic pollen monitoring, 1- to 3-hour values spread over the day are reported online with a 1- to 5-hour delay (the time depends on the instrument). Also, these rapid online data open new possibilities for next-day pollen predictions [55]. 

However, symptom severity correlates even more strongly with the concentration of allergens released from pollen grains than with pollen counts themselves [56], as demonstrated for Phl p 5 (grass pollen) in the UK [57]. Still, pollen counts are a good substitute parameter to explain allergic symptoms of allergic individuals as pollen are the only, although variable, source of these allergens. Because the direct measurement of airborne allergen concentrations is technically demanding and time-consuming, pollen counting remains a rapid, inexpensive, and practical alternative. 

AI in pollen recognition

Currently, several automated pollen monitoring systems using AI are capable of sampling, analyzing, and identifying airborne pollen and spores in near real time or real time. A comparative performance study conducted in Central Europe (Munich, Germany) evaluated multiple instruments by comparing their measurements with the mean values obtained from 4 co-located Hirst-type traps. Averaging the 4 manual samplers helped reduce uncertainties inherent to the manual counting method. From this comparison, 2 reliable automated devices emerged: the Hund BAA500 (Helmut Hund, Wetzlar, Germany), based on image recognition (Figure 1b), and the Swisens Poleno Jupiter, which combines holographic imaging with fluorescence spectral analysis (Figure 1c) [58]. 

For the Hund BAA500, pollen classification is performed using DL models based on CNNs [54, 59, 60, 61]. An important advantage of this system is the possibility of expert validation: all captured pollen images are stored online and can be re-examined individually by aerobiological experts, allowing human-in-the-loop verification of the automated classifications (https://validation.pollenscience.eu). 

The Swisens Poleno Jupiter system identifies airborne particles using holographic imaging combined with fluorescence spectral analysis. Its classification algorithm likewise relies on DL CNN-based methods [62]. In contrast to the Hund BAA500, this system provides fully automated online reporting, enabling faster data availability but offering fewer options for manual validation of individual particle classifications. 

Network with automatic devices

In Europe, there are only a few networks of automatic pollen monitors. The largest one is based on image recognition and will use about 28 Hund BAA500 devices and includes 8 automatic monitors (ePIN Network) in Bavaria, Germany, operational since 2019 [63]. The network has been expanding since 2025 under the leadership of the new German Weather Service into a Germany-wide network. Another major network is based in Switzerland (SwissPollen network) with 15 automatic monitors using the Swisens Poleno [62]. 

Pollen forecast using AI

Once the airborne concentration of allergenic pollen has been measured and delivered online to the public, the next step in supporting individuals with pollen allergies is pollen forecasting. The most widely used forecasting approaches rely on numerical atmospheric dispersion models, such as SILAM [64] and ICON-ART [55]. These models enable pollen forecasts at the European scale, but currently include only a limited number of allergenic pollen taxa [65]. More recently, ML-based approaches have been developed to improve pollen forecasting accuracy. Such models have been applied to predict airborne concentrations of *Ambrosia*, birch, *Olea*, and grass pollen, as well as *Quercus*, Cupressaceae, Urticaceae, and *Alternaria* spores [66, 67, 68, 69, 70, 71, 72]. These approaches already provide relatively accurate predictions at the local scale, although scaling such models to larger geographic regions remains challenging. 

### Conclusion 5 

Integrating real-time pollen data with AI-based meteorological and dispersion models is expected to improve forecast accuracy and exposure assessment. The distinct morphology of pollen enables reliable AI-based image recognition and automated analysis. Despite higher initial costs than Hirst-type traps, reduced manual labor may improve cost-effectiveness. Ongoing technological advances are likely to support broader implementation, benefiting research, and clinical allergy management. 

**4.1.2. Hymenoptera monitoring and classification**

AI, particularly DL-based image recognition systems, has substantially improved automated insect classification. Most published work to date has emerged from agricultural, ecological, or taxonomic research rather than from a clinical or allergological perspective [73, 74, 75, 76, 77, 78, 79, 80]. Nevertheless, these technological advances are directly relevant to allergology, particularly for the identification of medically important Hymenoptera species. 

The dominant methodological approach involves CNNs trained on annotated image datasets [73, 74, 75, 76, 77, 78]. High species-level classification performance has been demonstrated across multiple taxa. For example, DL models achieved 91.6% accuracy for bumble bee species identification [79], while automated hornet detection systems reported precision–recall F1 scores ≥ 0.99 for invasive *Vespa velutina* detection [76], highlighting a favorable relation of precision and recall. 

Real-time monitoring platforms integrating object detection and tracking demonstrated an average precision of 89% in field-based insect classification [77], and hybrid detection–tracking approaches achieved honeybee tracking rates of 86.6% in complex outdoor environments [73]. Embedded hive-monitoring systems based on YOLO (You Only Look Once), a fast architecture for object detection, reported F1 scores of 0.95 for honey bee detection and 96.28% tracking accuracy [75]. Beyond visual classification, complementary AI-supported approaches include morphometric and genomic methods. Geometric morphometrics discriminated honeybee subspecies with 84.9% accuracy at the individual wing level [74], while image-based neural network classification of parasitoid wasps achieved an average genus-level accuracy of 96% [78]. At the molecular level, whole-genome resequencing enabled the delineation of a previously undescribed *Apis mellifera* subspecies, demonstrating the role of genomic data in high-resolution taxonomic validation [80]. 

Collectively, these studies demonstrate expert-level classification performance under controlled and semi-field conditions. However, their primary focus remains agricultural monitoring, biodiversity research, and invasion biology rather than clinical allergology. Future translational efforts integrating AI systems for point-of-care classification and epidemiological surveillance may enable more precise environmental risk stratification and aid diagnostics for venom-allergic individuals [73, 74, 75, 76, 77, 78, 79, 80]. 

### Conclusion 6 

AI, particularly DL-based image recognition methods such as CNNs, has significantly advanced automated insect classification. Emerging translational applications integrating point-of-care AI with epidemiological surveillance may enhance environmental risk stratification and support the diagnosis of venom allergy. 

### 4.2. Clinical decision support systems 

Clinical decision support systems (CDSS) are digital systems designed to support clinical tasks and assist decision-making processes [81] (see Chapter 2). CDSS are already established in several areas of clinical practice, particularly in image-based diagnostics such as melanoma detection in dermatology [82]. 

Increasingly, CDSS are being explored as potent tools in allergology [81, 83]. 

The translation of CDSS into routine clinical practice in allergology is currently evolving and novel applications illustrate their potential for different clinical tasks, supporting diagnosis, risk stratification, and therapeutic decision-making. 

**4.2.1. Medical history/data analysis**

A potential field of application for CDSS lies in the partial automation of history-taking tasks. 

By efficiently processing large volumes of data, CDSS may contribute to substantial time savings and facilitate the structured collection of patient information, including medical history and relevant environmental factors. Conceptually, CDSS consist of three core components: data input, a processing algorithm or underlying AI, and the reporting of results [84]. 

At the level of AI-based processing, however, interpretation of results may be challenging due to the opacity of complex models. Approaches within the field of explainable artificial intelligence aim to address this limitation [85, 86]. 

Even today, AI-enhanced history-taking tools can be used in clinical practice, for example to handle incoming patient calls or to assist in documenting and transcribing patient histories. However, despite their increasing sophistication, these technologies remain imperfect, and require human oversight [87]. 

**4.2.2. Diagnostics/prospective information**

Improvements in medical diagnostics are particularly evident when AI is combined with human clinical assessment, compared with purely human-based evaluation. In specific domains, such as melanoma detection, AI systems already achieve diagnostic performance that equals or even exceeds individual human expertise, especially when used in conjunction with physician judgment [88]. In this context, CDSS may provide information that supports earlier recognition, risk stratification, and diagnostic confidence. CDSS can broadly support the interpretation of IgE microarrays for allergy diagnostics [83] and the etiological diagnosis of pollen allergy with AIT prescription [89]. A large-scale project for an AI-driven global atlas of IgE antibody responses to environmental antigens in humans has also been initiated [90], based on the use of IgE microarrays in patients with IgE-mediated allergies. 

**4.2.3. Therapeutic decision support**

Therapeutic decision support represents a core clinical application area of CDSS. ML-based CDSS can identify patients who may benefit from therapy modification based on sensitization profiles, biomarkers, demographic characteristics, prior medication, or disease course. In addition, such systems may alert physicians to newly available therapeutic options or recent extensions of regulatory approvals that may not previously have been considered for an individual patient [91]. In allergology, CDSS have been developed to assist in the prescription of AIT to this end [89] as well as for day-to-day pharmacological management and short-term prevention of seasonal allergic rhinitis [92]. 

Further CDSS for health surveillance of pollen allergy have been proposed using a crowdsourced digital application based on a participatory and person-centered digital medicine approach utilizing volunteered geographic information, supporting personalized short-term prediction of allergic symptoms in patients with hay fever [93]. 

In current applications of CDSS in allergology, their supportive character remains central, as these systems are intended to complement clinical decision-making rather than replace medical professionals. However, the role of CDSS may evolve in the future of allergology, with AI increasingly discussed as a “collaborative partner” rather than merely a support tool [94]. 

**4.2.4. Practical applications**

CDSS can be used to structure and search large databases for specific information, including multimodal clinical data, assisting especially at bottlenecks in precision medicine [81], and more generally, in complex clinical tasks [95]. Potential fields of application in allergology are detailed in Table 1. 

**4.2.5. Outlook**

Reliable use of CDSS in allergology requires models whose training reflects the linguistic and structural characteristics of medical texts in different languages as well as variations in national regulatory frameworks. At the same time, the systematic transfer of established ML approaches from other medical disciplines should be evaluated to support the development of allergology-specific, task-focused, and agentic CDSS. 

In allergology, the development of guideline-based and retrieval-augmented generation-supported systems, together with companion tools endorsed by professional societies, could serve as a model for prognostic decision support in other medical fields. For broader clinical adoption, readily deployable out-of-the-box CDSS tools are required to enable rapid translation into routine allergological care. 

A human-in-the-loop approach remains essential, particularly for complex and high-stakes clinical tasks. This perspective aligns with emerging approaches that deploy AI in agentic networks, in which specialized agents are optimized for specific tasks rather than relying on a single, less specific multipurpose system [29, 102]. 

### Conclusion 7 

CDSS are digital tools designed to support clinical tasks and decision-making. Their integration into routine allergological practice is evolving, with emerging applications demonstrating potential for diagnosis, risk stratification, and therapeutic decisions. 

Reliable implementation requires models trained on linguistic and structural characteristics of medical texts in different languages and aligned with variations in national regulatory frameworks. A human-in-the-loop approach remains essential, particularly for complex, high-stakes tasks, consistent with emerging AI concepts based on task-specific, agentic networks rather than general-purpose systems. 

### 4.3. Patient education and self-management support 

Supportive AI tools for patients include chatbots, connective online platforms, mobile applications, wearables, and smart (sensor) technologies. In the past decade, a rising number of digital solutions has been developed and evaluated [103]. In the following, we focus exclusively on those implementing ML or other aspects of AI. 

**Conversational agents (chatbots) to support patient self-management**

Despite the current diversity of available chatbots, ChatGPT (Open AI) gains outstanding attention in the public as well as in the scientific community. Most individuals in Germany made their first experience with generative AI in the form of an LLM, mainly ChatGPT [104, 105]. Consequently, this chatbot has been most frequently evaluated in the medical context of allergology. A recent performance assessment of ChatGPT4 across 120 interdisciplinary questions around allergies resulted in a reasonably good accuracy of the model compared to expert opinion (mean accuracy 4.1/5, standard deviation: 0.78, range: 1 – 5). Yet, the chatbot delivered erroneous information related to a case of pediatric food allergy, which could have resulted in a life-threatening risk, suggesting a need for human (expert) supervision [22]. Studies on the performance of ChatGPT on queries regarding atopic dermatitis [106], allergic rhinitis, chronic rhinosinusitis [107], chronic urticaria [108], and oral immunotherapy [109] showed a good performance and underline the potential of LLM technology to deliver medical information independently of the availability of medical staff. 

A specific performance evaluation of ChatGPT-4 regarding 24 questions on allergen immunotherapy also showed a solid correlation between the LLM and expert opinion [36], but in line with others [110], the authors underline a lack of transparency and readability, especially for users without previous knowledge on the topic or academic education. Particularly the risks of limited transparency and potentially life-threatening accuracy flaws have been addressed by Aulenbacher et al. [31], who created a chatbot for chronic spontaneous urticaria queries based on pre-selected sources from a systematic literature search. This approach led to a significantly higher average accuracy (94.6%) compared to ChatGPT-3.5 (42.6%) and ChatGPT-4o (85.7%). An app-based intervention for patients with grass pollen allergy demonstrated that AI-based symptom forecasting features improved patient self-management and reduced symptom burden [92]. 

In summary, conversational agents have the potential to play a significant role in information delivery, patient education, and guidance [111, 112]. However, transparency, human oversight and careful source selection are needed to ensure patient safety, as also discussed in chapter 4.2. 

**Tackling treatment adherence with AI tools**

As acknowledged by the WHO, “increasing the effectiveness of adherence interventions may have a far greater impact on the health of the population than any improvement in specific medical treatments” [113]. The logical consequence is the development and adaptation of AI tools also for this purpose. Digital tools in general have been shown to be effective measures to increase adherence [114, 115]. However, research on AI tools in this specific area is still scarce [116]; No AI-enabled medical devices have been approved by the FDA in the field of allergy so far [117]. To target patients at high risk of low adherence, several predictive ML models have been evaluated to enable timely, personalized interventions. Applications range from subcutaneous immunotherapy [118], over asthma medication [119], to adolescents receiving personalized motivational messages or gamified reminders if they forget to take medication or carry their adrenaline autoinjector [120]. 

Lv et al. [119] performed a randomized controlled study proving that a mobile application-assisted intervention could improve adherence to anti-asthmatic medication in 152 children (6 – 11.9 years old), using medication reminders, exacerbation alerts, providing treatment recommendations, and facilitating instant communication with healthcare providers. Another example is the specifically developed PollDi App [92] for grass pollen-allergic patients that was tested in a stratified, controlled intervention study. The working group was able to show a significant improvement of nasal and conjunctival allergic symptoms of participants with a full version of the smartphone app vs. limited access. Especially the availability of near real-time pollen data and forecasts led to increased self-medication and thereby reduced symptom severity. A Chinese randomized controlled trial was able to show that a smartwatch-based medication detection and reminder tool [121] significantly improved medication intake and relieved allergic rhinitis symptoms in the study group. Further, AI-driven wheeze detectors [122] and digital inhalers [123] have been identified as potentially valuable tools to support the correct application of short-acting beta-agonist and anti-inflammatory asthma medication, both in terms of timing and inhalation technique. 

Among patients suffering from atopic dermatitis and chronic urticaria, changes in skin condition require adaptations of treatment plans, making adherence challenging for a proportion of patients [124]. 

Future fields of AI application in the area of atopic dermatitis involve systems assessing eczema control, thus recognizing deterioration of skin status or flares and encouraging patients to seek advice from their health care provider. Assessment of itch intensity via electronic questionnaires can also add to a better recognition of disease severity via AI-driven systems [125]. 

Although applications improving adherence to therapy (both topical and systemic) would possibly show positive effects on eczema control and patients’ quality of life, there is no disease-specific scientific evidence on the role of AI technologies to improve treatment adherence. However, several publications report the feasibility of monitoring drug intake using smart pill bottles [126], an approach that could be expanded to topical skin treatment dispensers. However, it remains to be discussed if this approach is favorable for the patient–doctor/nurse relationship. 

### Conclusion 8 

In patient education and self-management, only a few digital applications in the field of allergy include advanced AI models. Current evidence is limited by mixed quality and a lack of standardized evaluation methods for both AI tools and adherence. However, AI has potential to improve these patient empowerment tools, particularly among those in underserved areas, or those at high risk of non-adherence. 

## Authors’ contributions 

Oliver Pfaar: writing, conceptualization, development of design and methodology, review and editing. Stephan Traidl: writing, conceptualization, development of design and methodology, review and editing. Friederike Bärhold: writing, review, and editing. Sven Becker: writing, review, and editing. Christoph Bergmann: writing, conceptualization, review, and editing. Jeroen Buters: writing, visualization, review, and editing. Stephanie Dramburg: writing, conceptualization, review, and editing. Wojciech Francuzik: writing, conceptualization, review, and editing. Michael Gerstlauer: conceptualization, review, and editing. Eckard Hamelmann: writing, review, and editing. Camilo José Hernandez Toro: writing, conceptualization. Ludger Klimek: review and editing. Lars Lange: writing, visualization, review, and editing. Paolo M. Matricardi: conceptualization, review, and editing. Laura Pfeffer: writing, review, and editing. Mariel Suarez-Suarez: writing, visualization, review, and editing. Philipp Utz: writing, review, and editing. Martin Wagenmann: drafting, writing, conceptualization, review, and editing. Margitta Worm: writing, review, and editing. Mehrak Yoosefi: writing, conceptualization, review, and editing. Sebastian Seurig: writing, management and coordination (co-lead), conceptualization, development of design and methodology, review, and editing. Sonja Mathes: writing, management and coordination (co-lead), conceptualization, development of design and methodology, review, and editing. 

## Funding 

There was no external funding for this study. 

## Conflict of interest 

Oliver Pfaar reports grants and/or personal fees and/or travel support from AEDA, Alfried Krupp Krankenhaus, ALK-Abelló, Allergopharma, Almirall, Altamira Therapeutics, ASIT Biotech, AstraZeneca, Bencard Allergie GmbH/Allergy Therapeutics, Biologixpharma, Blueprint, Breazy Health, Cliantha, Deutsche AllergieLiga e.V., Cogitando GmbH/medcram, Deutsche Forschungsgemeinschaft, Dustri-Verlag, ECM Expro&Conference Management GmBH, Forum für Medizinische Fortbildung, Georg-Thieme-Verlag, GSK, HAL Allergy Holding B.V./HAL Allergie GmbH, Inmunotek, Ingress Health, Institut für Disease Management, IQVIA Commercial, Japanese Society of Allergology, Königlich Dänisches Generalkonsulat, Laboratorios LETI/LETI Pharma, Lilly, Lofarma, Medcram/Cogitando, Medizinische Hochschule Hannover, med update europe GmbH, Meinhardt Congress GmbH, Novartis, Paul-Ehrlich-Institut, Paul-Martini-Stiftung, PneumoLive, Pohl-Boskamp, Procter & Gamble, Red Maple Trials Inc., Regeneron, RG Aerztefortbildung, ROXALL Medizin, Sanofi Aventis, Sanofi Genzyme, Springer Publisher, Stallergenes Greer, streamedup! GmbH, Technical University Dresden, John Wiley & sons publishers, Wort & Bild Verlag, Verlag ME, Gemeinnütziger Verein zur Förderung der Allergiefortbildung (VFAF), all outside the submitted work. Oliver Pfaar is board member of the ENT section of the European Academy of Allergy and Clinical Immunology (EAACI) and member of the extended board of directors of the German Society of Allergy and Clinical Immunology (DGAKI); coordinator, main- or co-author of different position papers and guidelines in rhinology, allergology and allergen-immunotherapy; and he is Editor-in-Chief of Clinical Translational Allergy. 

Stephan Traidl has received honoraria for lectures or scientific advice from AbbVie, ALK, Almirall, Galderma, Janssen/JNJ, LEO Pharma, Lilly, Novartis, and Regeneron/Sanofi. 

Friederike Bärhold received speaker honoraria or advisory board fees from GSK, Bencard, AstraZeneca, Leti, and travel reimbursement from HAL Allergy. FB is involved in research sponsored by Astra Zeneca, Sanofi, GSK, all outside the here-submitted work. 

Sven Becker has received lecture fees and participated in advisory boards of Allergopharma, Allergy-Therapeutics, ALK-Abelló, Ambu, AstraZeneca, Bencard Allergie, GSK, HAL Allergie, MSD, Sanofi, Smart Reporting, Stallergenes; and received research grants from Allergy-Therapeutics, BMBF, Akribion, Polyrizon, BRAIN AG, Pari, and Altamira AG. 

Christoph Bergmann received honoraria for presentations or advisory from Allergy Therapeutics, Bencard, Astra Zeneca, GSK, HAL Allergy, and SCS. 

Jeroen Buters has received honoraria for lectures from ALK, Allergopharma, Stallergenes, AEDA, and DGAKI. Other research funding was obtained from DFG, EU, and the Bavarian Government LGL. 

Stephanie Dramburg reports research funding from Thermo Fisher Scientific, speaker´s fees from Allergopharma, LETI, Bencard, dbv Technologies, OMRON Healthcare, Stallergenes, ALK, Siemens Healthineers, and is member of an advisory board for Bencard, as well as member of a data safety monitoring board for Allergy Therapeutics. 

Wojciech Francuzik declares no COI. 

Michael Gerstlauer is involved in studies with Allergopharma, Bencard Allergie, Boehringer, InfectoPharm, Stallergenes-Greer, has served at consulting for and received speaker honoraria from ALK-Abelló, Allergopharma, Bencard Allergie, Leti Pharma, Meda Pharma, Sanofi, Stallergenes-Greer, is part of specialist societies AGPAS, GPAU, GPP, DGKJ, EAACI, ERS, and is involved in AWMF guidelines for Allergen Immunotherapy, Insect Venom Allergy, Allergy Prevention, Severe Bronchial Asthma In Childhood, Allergic Rhinoconjunctivitis, S2k Bronchial Asthma, National Disease Management Guideline Bronchial Asthma, and the EAACI guideline on Allergen Immunotherapy. 

Eckard Hamelmann declares no COI. 

Camilo José Hernandez Toro declares no COI. 

Ludger Klimek has received research grants from Allergy Therapeutics/Bencard, United Kingdom/Germany; ALK-Abelló, Denmark; Allergopharma, Germany; ASIT Biotech, Belgium; AstraZeneca, Sweden; Bionorica, Germany; Biomay, Austria; Boehringer Ingelheim, Germany; Circassia, USA; Stallergenes, France; Cytos, Switzerland; Curalogic, Denmark; HAL, the Netherlands; Hartington, Spain; Lofarma, Italy; Viatris/Mylan, USA; Novartis, Switzerland; Leti, Spain; Roxall, Germany; GlaxoSmithKline (GSK), UK; and Sanofi, France, and/or has served as a speaker or worked as a consultant for the aforementioned pharmaceutical companies. LK is President of the Ärzteverband Deutscher Allergologen (AeDA), Vice President of the German Academy for Allergy and Environmental Medicine (DAAU), and a member of the Governance and NAIS Committees of the European Academy of Allergy and Clinical Immunology (EAACI); he is also editor-in-chief of *AllergoJournal* and *AllergoJournal International*. 

Lars Lange has received lecture fees and participated in advisory boards from Stallergenes, Allergopharma, Infectopharm, HAL Allergy, Thermo Fisher Scientific, DBV technologies, Novartis, Nestle Nutritional Institute, Nutricia, Sanofi, all outside the submitted work. 

Paolo M. Matricardi reports research support, speaker and/or consultancy fees from Allergopharma, Euroimmun, Hycor, MAD-X, Omron Healthcare, Stallergenes-Greer, Thermo Fisher Scientific, research support from TPS software production, speaker and/or consultancy fees from ALK-Abellò, Angelini, Bencard Allergie, HAL, all outside the submitted work. 

Laura Pfeffer declares no COI. 

Mariel Suarez-Suarez declares no COI. 

Philipp Utz has received honoraria for lectures or scientific advice from Aimmune, Allergopharma, ALK-Abelló, Berlin-Chemie, DAAB, Nutricia, PARI, and Sanofi-Regeneron. 

Martin Wagenmann has received lecture fees and participated in advisory boards of Allergopharma, ALK-Abelló, AstraZeneca, Celltrion, CSL Behring, Genzyme, GSK, HAL Allergie, MSD, NeilMed, Regeneron, Sanofi, Stallergenes; and received research grants from ALK-Abelló, AstraZeneca, EU, GSK, Novartis, Regeneron, Sanofi, Takeda. 

Margitta Worm reports honoraria and/or consulting fees from AbbVie, ALK-Abelló, Allergopharma, Almirall, Amgen, Boehringer Ingelheim, DBV Technologies, Genzyme, Kymab, LEO Pharma, Eli Lilly, Mylan (Viatris), Novartis, Pfizer, Regeneron Pharmaceuticals Inc., Sanofi, Stallergenes Greer. 

Mehrak Yoosefi declares no COI. 

Sebastian Seurig reports personal financial interests: ALK-Abelló (consulting, advisory board); AstraZeneca (lectures/training); Allergopharma (lectures); Bencard Allergy (advisory board). 

Sonja Mathes received speaker honoraria from Thermo Fisher, ALK-Abelló, AstraZeneca and Bencard and travel reimbursement by ALK-Abelló and Bencard. 

## EQUATOR AGREE II 

Compliance with EQUATOR AGREE II statement. 

**Figure 1:**
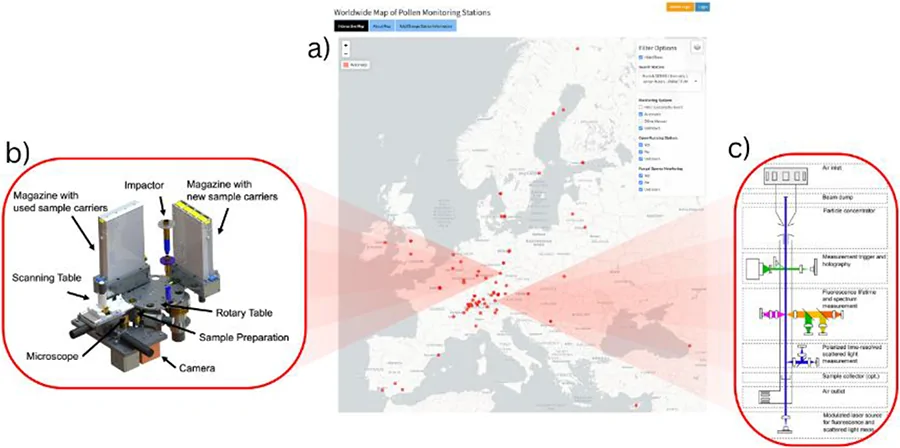
a: Map of pollen monitoring stations. Red dots indicate automatic pollen monitors. b: Automatic pollen monitor based on image recognition (Hund BAA500). c: Automatic pollen monitor based on holographic imaging and fluorescence spectra (Swisens Poleno Jupiter, Swisens AG, Emmen, Switzerland). Both instruments use DL CNN algorithms for particle classification. See www.ZAUM-online.de, accessed March 2026.

**Table 1.** Emerging applications of clinical decision support systems in allergology.

**Clinical field**	**Emerging CDSS applications**	**Applicability and clinical benefit in allergology**
Pediatric practice	Predominantly rule-based CDSS for triage and diagnostic support, with emerging AI-based CDSS for acute care risk stratification (e.g., pneumonia detection [96]). AI-enhanced assistance tools for communication and clinical documentation, including telephone triage and speech-to-text systems [97].	Support of triage and prioritization in pediatric emergency and outpatient settings; assistance in asthma phenotyping and endotyping; structured assessment of symptom patterns and exacerbation risk; reduction of documentation burden and optimization of clinical workflows.
Dermatology/allergological dermatology	Emerging CDSS for monitoring inflammatory skin diseases [98]; AI-based tools for precision and semi-automated skin-prick testing [32].	Reduction of interobserver variability; prioritization of cases; adjunctive support for monitoring disease activity in atopic dermatitis and chronic urticaria.
ENT allergology (rhinology/conjunctiva)	Emerging CDSS for structured symptom assessment, interpretation of nasal and conjunctival provocation tests, integration of clinical, endoscopic, and laboratory data, etiological diagnosis of seasonal allergic rhinitis, allergen immunotherapy prescription, day-to-day pharmacological management, and short-term prevention of seasonal allergic rhinitis [89].	Support for indication, standardization, and interpretation of nasal and conjunctival allergen provocation testing; improved reproducibility and safety; assistance in the etiological diagnosis of allergic rhinitis and differentiation from non-allergic rhinitis; facilitation of guideline-adherent diagnostic pathways.
Cross-specialty clinical tasks	CDSS for real-time data integration, structured anamnesis, and interpretation of complex laboratory data (e.g., IgE microarrays and molecular allergy diagnostics) [99]. AI-enhanced assistance tools for communication and clinical documentation, including telephone triage and speech-to-text systems [100].	Support for standardization of allergological diagnostic work-ups; facilitation of interdisciplinary clinical decision-making; improved consistency in complex cases (e.g., polysensitization or multimorbidity); support for precision-oriented diagnostic and therapeutic strategies.
Administrative and organizational processes	AI-supported CDSS for diagnostic coding and billing [101].	Optimization of practice organization; reduction of administrative workload; improved coding accuracy; indirect quality gains through reallocation of clinician time toward patient care.

CDSS = clinical decision support systems; AI = artificial intelligence.

## References

[1] Garcia-Aymerich J Benet M Saeys Y Pinart M Basagaña X Smit HA Siroux V Just J Momas I Rancière F Keil T Hohmann C Lau S Wahn U Heinrich J Tischer CG Fantini MP Lenzi J Porta D Koppelman GH Phenotyping asthma, rhinitis and eczema in MeDALL population-based birth cohorts: an allergic comorbidity cluster. Allergy. 2015; 70: 973–984. 10.1111/all.12640 25932997

[2] Cunha F Amaral R Jacinto T Sousa-Pinto B Fonseca JA A Systematic Review of Asthma Phenotypes Derived by Data-Driven Methods. Diagnostics (Basel). 2021; 11:644. 10.3390/diagnostics11040644 PMC8066118 33918233

[3] Lötvall J Akdis CA Bacharier LB Bjermer L Casale TB Custovic A Lemanske RF Wardlaw AJ Wenzel SE Greenberger PA Asthma endotypes: a new approach to classification of disease entities within the asthma syndrome. J Allergy Clin Immunol. 2011; 127: 355–360. 10.1016/j.jaci.2010.11.037 21281866

[4] Maintz L Welchowski T Herrmann N Brauer J Kläschen AS Fimmers R Schmid M Bieber T Schmid-Grendelmeier P Traidl-Hoffmann C Akdis C Lauener R Brüggen MC Rhyner C Bersuch E Renner E Reiger M Dreher A Hammel G Luschkova D Machine Learning-Based Deep Phenotyping of Atopic Dermatitis: Severity-Associated Factors in Adolescent and Adult Patients. JAMA Dermatol. 2021; 157: 1414–1424. 10.1001/jamadermatol.2021.3668 PMC8581798 34757407

[5] Howard R Fontanella S Simpson A Murray CS Custovic A Rattray M Component-specific clusters for diagnosis and prediction of allergic airway diseases. Clin Exp Allergy. 2024; 54: 339–349. 10.1111/cea.14468 38475973

[6] Kilanowski A Thiering E Wang G Kumar A Kress S Flexeder C Bauer CP Berdel D von Berg A Bergström A Gappa M Heinrich J Herberth G Koletzko S Kull I Melén E Schikowski T Peters A Standl M Allergic disease trajectories up to adolescence: Characteristics, early-life, and genetic determinants. Allergy. 2023; 78: 836–850. 10.1111/all.15511 36069615

[7] Seurig S Traidl S Mathes S. The Role of Artificial Intelligence in Modern Allergology: A Review of Applications in Diagnosis, Prediction, and Management. JEADV Clin Pract. 2026; e70297.

[8] Kaczmarczyk R Lasser T Biedermann T Ring J Zink A Revealing clinically relevant specific IgE sensitization patterns in Hymenoptera venom allergy with dimension reduction and clustering. World Allergy Organ J. 2023; 16: 100820. 10.1016/j.waojou.2023.100820 PMC10562856 37822702

[9] Fomina DS Lebedkina MS Nikitina EA Dushkin AD Chernov AA Yukhnovskaya YD Evsegneeva IV Karaulov AV Lysenko MA Artificial intelligence tools for molecular analysis of sensitization profiles in patients with atopic dermatitis and bronchial asthma. Russ J Biotherapy. 2024; 23: 26–39.

[10] Moreno EM Moreno V Laffond E Gracia-Bara MT Muñoz-Bellido FJ Macías EM Curto B Campanon MV de Arriba S Martin C Davila I Usefulness of an Artificial Neural Network in the Prediction of β-Lactam Allergy. J Allergy Clin Immunol Pract. 2020; 8: 2974–2982. 10.1016/j.jaip.2020.07.010 32702519

[11] Trubiano JA Vogrin S Chua KYL Bourke J Yun J Douglas A Stone CA Yu R Groenendijk L Holmes NE Phillips EJ Development and Validation of a Penicillin Allergy Clinical Decision Rule. JAMA Intern Med. 2020; 180: 745–752. 10.1001/jamainternmed.2020.0403 PMC7076536 32176248

[12] Inglis JM Bacchi S Troelnikov A Smith W Shakib S Automation of penicillin adverse drug reaction categorisation and risk stratification with machine learning natural language processing. Int J Med Inform. 2021; 156: 104611. 10.1016/j.ijmedinf.2021.104611 34653809

[13] Gryak J Georgievska A Zhang J Najarian K Ravikumar R Sanders G Schuler CF Prediction of pediatric peanut oral food challenge outcomes using machine learning. J Allergy Clin Immunol Glob. 2024; 3: 100252. 10.1016/j.jacig.2024.100252 PMC11090861 38745865

[14] Kuniyoshi Y Tokutake H Takahashi N Kamura A Yasuda S Tashiro M Machine learning approach and oral food challenge with heated egg. Pediatr Allergy Immunol. 2021; 32: 776–778. 10.1111/pai.13433 33326635

[15] DunnGalvin A Daly D Cullinane C Highly accurate prediction of food challenge outcome using routinely available clinical data. J Allergy Clin Immunol. 2011; 127: 633–639. 10.1016/j.jaci.2010.12.004 21377032

[16] Mormile I Granata F Detoraki A Pacella D Della Casa F De Rosa F Romano A de Paulis A Rossi FW Predictive Response to Immunotherapy Score: A Useful Tool for Identifying Eligible Patients for Allergen Immunotherapy. Biomedicines. 2022; 10: 971. 10.3390/biomedicines10050971 PMC9138211 35625708

[17] Wu JJ Hong CH Merola JF Gruben D Güler E Feeney C Bhambri A Myers DE DiBonaventura M Predictors of nonresponse to dupilumab in patients with atopic dermatitis: A machine learning analysis. Ann Allergy Asthma Immunol. 2022; 129: 354–359. 10.1016/j.anai.2022.05.025 35640774

[18] Seurig S Traidl S Mathes S Machine Learning – was steckt dahinter? Allergologie. 2025; 48: 317–323.

[19] Morone G De Angelis L Martino Cinnera A Carbonetti R Bisirri A Ciancarelli I Iosa M Negrini S Kiekens C Negrini F Artificial intelligence in clinical medicine: a state-of-the-art overview of systematic reviews with methodological recommendations for improved reporting. Front Digit Health. 2025; 7: 1550731. 10.3389/fdgth.2025.1550731 PMC11920125 40110115

[20] Matricardi PM Bousquet J Clinical Decision Support Systems for Allergic Diseases and Asthma In: Digital Allergology. Health Informatics. Cham: Springer Nature; 2025; 95-117.

[21] Cestonaro C Delicati A Marcante B Caenazzo L Tozzo P Defining medical liability when artificial intelligence is applied on diagnostic algorithms: a systematic review. Front Med (Lausanne). 2023; 10: 1305756. 10.3389/fmed.2023.1305756 PMC10711067 38089864

[22] Mathes S Seurig S Bluhme F Beyer K Heizmann F Wagner M Neugärtner I Biedermann T Darsow U ChatGPT Performance on 120 Interdisciplinary Allergology Questions-Systematic Evaluation With Clinical Error Impact Assessment for Critical Erroneous AI-Guided Chatbot Advice. J Allergy Clin Immunol Pract. 2025; 13: 1350–1357. 10.1016/j.jaip.2025.03.030 40157421

[23] Raman R Pattnaik D Hughes L Nedungadi P Unveiling the dynamics of AI applications: A review of reviews using scientometrics and BERTopic modeling. J Innov Knowl. 2024; 9: 100517.

[24] Bajwa J Munir U Nori A Williams B Artificial intelligence in healthcare: transforming the practice of medicine. Future Healthc J. 2021; 8: e188–e194. 10.7861/fhj.2021-0095 PMC8285156 34286183

[25] Brouwers MC Kerkvliet K Spithoff K The AGREE Reporting Checklist: a tool to improve reporting of clinical practice guidelines. BMJ. 2016; 352: i1152. 10.1136/bmj.i1152 PMC5118873 26957104

[26] Bzdok D Altman N Krzywinski M Statistics versus machine learning. Nat Methods. 2018; 15: 233–234. 10.1038/nmeth.4642 PMC6082636 30100822

[27] Heilbroner SP Miotto R Deep Learning in Medicine. Clin J Am Soc Nephrol. 2023; 18: 397–399. 10.2215/CJN.0000000000000080 PMC10103223 36735512

[28] Ghassemi M Oakden-Rayner L Beam AL The false hope of current approaches to explainable artificial intelligence in health care. Lancet Digit Health. 2021; 3: e745–e750. 10.1016/S2589-7500(21)00208-9 34711379

[29] van Breugel M Greenhawt M Eguiluz-Gracia I Torres Jaén MJ Anagnostou A Koppelman GH Artificial intelligence in allergy and immunology: Recent developments, implementation challenges, and the road toward clinical impact. J Allergy Clin Immunol. 2026; 157: 295–306. 10.1016/j.jaci.2025.08.022 40925528

[30] Yarin Y Kalaitzidou A Bodrova K Mösges R Kalaidzidis Y Validation of AI-based software for objectification of conjunctival provocation test. J Allergy Clin Immunol Glob. 2023; 2: 100121. 10.1016/j.jacig.2023.100121 PMC10509841 37779521

[31] Aulenbacher F Gutsche A Bihlmaier B Bonnekoh H Cherrez-Ojeda I Fluhr JW Kolkhir P Magerl M Metz M Pyatilova P Siebenhaar F Zuberbier T Neisinger S Development and Reliability Assessment of an Artificial Intelligence-Driven Urticaria Support (AIDUS) Chatbot. J Allergy Clin Immunol Pract. 2025; 13: 2960–2967. 10.1016/j.jaip.2025.07.047 40780383

[32] Seys SF Hox V Chaker AM De Greve G Lemmens W Poirrier AL Beckers E Daems R Diamant Z Dierickx C Hellings PW Huart C Jerin C Jorissen M Loeckx D Oscé H Roux K Thompson M Tombu S Uyttebroek S Artificial Intelligence (AI)-assisted readout method for the evaluation of skin prick automated test results. Nat Commun. 2025; 16: 8637. 10.1038/s41467-025-64334-w PMC12488876 41034189

[33] Dorszewski T Tětková L Jenssen R Hansen LK Wickstrøm KK From Colors to Classes: Emergence of Concepts in Vision Transformers In: Guidotti R, Schmid U, Longo L, (eds). Explainable Artificial Intelligence. Vol 2576. Communications in Computer and Information Science. Cham: Springer Nature; 2026; p.28-47.

[34] Milling M Rampp SDN Triantafyllopoulos A Plaza MP Brunner JO Traidl-Hoffmann C Schuller BW Damialis A Automating airborne pollen classification: Identifying and interpreting hard samples for classifiers. Heliyon. 2025; 11: e41656. 10.1016/j.heliyon.2025.e41656 PMC11782946 39897809

[35] Boldeanu M González-Alonso M Cucu H Burileanu C Maya-Manzano JM Buters JTM Automatic Pollen Classification and Segmentation Using U-Nets and Synthetic Data. IEEE Access. 2022; 10: 73675–73684.

[36] Cherrez-Ojeda I Zuberbier T Rodas-Valero G Sanchez J Rudenko M Dramburg S Demoly P Caimmi D Gómez RM Ramon GD Fouda GE Quimby KR Chong-Neto H Llosa OC Larco JI Monge Ortega OP Faytong-Haro M Pfaar O Bousquet J Robles-Velasco K Evaluation of the Quality and Reliability of ChatGPT-4’s Responses on Allergen Immunotherapy Using Validated Instruments for Health Information Quality Assessment. Clin Transl Allergy. 2025; 15: e70130. 10.1002/clt2.70130 PMC12665041 41319041

[37] For trustworthy AI, keep the human in the loop. Nat Med. 2025; 31: 3207–3207. 10.1038/s41591-025-04033-7 41073516

[38] Lu W Luu RK Buehler MJ Fine-tuning large language models for domain adaptation: exploration of training strategies, scaling, model merging and synergistic capabilities. NPJ Comput Mater. 2025; 11: 84.

[39] D’Amato G Vitale C Sanduzzi A Molino A Vatrella A D’Amato M Allergenic pollen and pollen allergy in Europe. In: Apple Academic Press; 2017. Accessed April 22, 2026. https://www.iris.unisa.it/handle/11386/4762888

[40] Falagiani P CRC Press; 1989.

[41] Biedermann T Winther L Till SJ Panzner P Knulst A Valovirta E Birch pollen allergy in Europe. Allergy. 2019; 74: 1237–1248. 10.1111/all.13758 30829410

[42] Skjøth CA Šikoparija B Jäger S EAN-Network. Pollen Sources. In: Sofiev M, Bergmann KC (eds). Allergenic Pollen. Dordrecht: Springer; 2013. p. 9-27.

[43] Liccardi G D’Amato M D’Amato G Oleaceae pollinosis: a review. Int Arch Allergy Immunol. 1996; 111: 210–217. 10.1159/000237370 8917115

[44] Vara A Fernández-González M Aira MJ Rodríguez-Rajo FJ Oleaceae cross-reactions as potential pollinosis cause in urban areas. Sci Total Environ. 2016; 542: 435–440. 10.1016/j.scitotenv.2015.10.107 26520268

[45] García-Mozo H Poaceae pollen as the leading aeroallergen worldwide: A review. Allergy. 2017; 72: 1849–1858. 10.1111/all.13210 28543717

[46] Hrabina M Peltre G Van Ree R Moingeon P Grass pollen allergens. Clin Exp Allergy Rev. 2008; 8: 7–11.

[47] Jaeger S Exposure to grass pollen in Europe. Clin Exp Allergy Rev. 2008; 8: 2–6.

[48] Buters JTM Antunes C Galveias A Bergmann KC Thibaudon M Galán C Schmidt-Weber C Oteros J Pollen and spore monitoring in the world. Clin Transl Allergy. 2018; 8: 9. 10.1186/s13601-018-0197-8 PMC5883412 29636895

[49] Oteros J Buters J Laven G Röseler S Wachter R Schmidt-Weber C Hofmann F Errors in determining the flow rate of Hirst-type pollen traps. Aerobiologia (Bologna). 2017; 33: 201–210.

[50] Suarez-Suarez M Maya-Manzano JM Clot B Graber M-J Sallin C Tummon F Buters J Accuracy of a hand-held resistance-free flowmeters for flow adjustments of Hirst-Type pollen traps. Aerobiologia (Bologna). 2023; 39: 143–148.

[51] Triviño MM Maya-Manzano JM Tummon F Clot B Grewling Ł Schmidt-Weber C Buters J Variability between Hirst-type pollen traps is reduced by resistance-free flow adjustment. Aerobiologia (Bologna). 2023; 39: 257–273.

[52] Buters J Clot B Galán C Gehrig R Gilge S Hentges F O’Connor D Sikoparija B Skjoth C Tummon F Adams-Groom B Antunes CM Bruffaerts N Çelenk S Crouzy B Guillaud G Hajkova L Seliger AK Oliver G Ribeiro H Automatic detection of airborne pollen: an overview. Aerobiologia (Bologna). 2024; 40: 13–37.

[53] Clot B Gilge S Hajkova L Magyar D Scheifinger H Sofiev M Bütler F Tummon F The EUMETNET AutoPollen programme: establishing a prototype automatic pollen monitoring network in Europe. Aerobiologia (Bologna). 2024; 40: 3–11.

[54] Oteros J Pusch G Weichenmeier I Heimann U Möller R Röseler S Traidl-Hoffmann C Schmidt-Weber C Buters JT Automatic and Online Pollen Monitoring. Int Arch Allergy Immunol. 2015; 167: 158–166. 10.1159/000436968 26302820

[55] Hoshyaripour GA Baer A Bierbauer S Bruckert D Brunner D Foerstner J Hamzehloo A Hanft V Keller C Klose M Kumar P Ludwig P Metzner E Muth L Pauling A Porz N Reddmann T Reißig L Ruhnke R Satitkovitchai K Seifert A Sinnhuber M Steiner M Versick S Vogel H Weimer M Werchner S Hoose C The Atmospheric Composition Component of the ICON modeling framework: ICON-ART version 2025.10. Geoscientific Model Development. 2026; 19: J 1645–1681.

[56] Durham SR Nelson HS Nolte H Bernstein DI Creticos PS Li Z Andersen JS Magnitude of efficacy measurements in grass allergy immunotherapy trials is highly dependent on pollen exposure. Allergy. 2014; 69: 617–623. 10.1111/all.12373 PMC4314686 24605984

[57] Fuertes E Jarvis D Lam H Davies B Fecht D Candeias J Schmidt-Weber CB Douiri A Slovick A Scala E Smith TEL Shamji M Buters JTM Cecchi L Till SJ Phl p 5 levels more strongly associated than grass pollen counts with allergic respiratory health. J Allergy Clin Immunol. 2024; 153: 844–851. 10.1016/j.jaci.2023.11.011 37995860

[58] Maya-Manzano JM Tummon F Abt R Allan N Bunderson L Clot B Crouzy B Daunys G Erb S Gonzalez-Alonso M Graf E Grewling Ł Haus J Kadantsev E Kawashima S Martinez-Bracero M Matavulj P Mills S Niederberger E Lieberherr G Towards European automatic bioaerosol monitoring: Comparison of 9 automatic pollen observational instruments with classic Hirst-type traps. Sci Total Environ. 2023; 866: 161220. 10.1016/j.scitotenv.2022.161220 36584954

[59] Farooq Q Oteros J Galán C Advancing in the pollen frontier: a comprehensive evaluation and meta-analysis of automatic pollen monitoring systems. Aerobiologia (Bologna). 2025; 41: 527–546.

[60] González-Alonso M Boldeanu M Koritnik T Gonçalves J Belzner L Stemmler T Gebauer R Grewling Ł Tummon F Maya-Manzano JM Ariño AH Schmidt-Weber C Buters J Alternaria spore exposure in Bavaria, Germany, measured using artificial intelligence algorithms in a network of BAA500 automatic pollen monitors. Sci Total Environ. 2023; 861: 160180. 10.1016/j.scitotenv.2022.160180 36403848

[61] Oteros J Weber A Kutzora S Rojo J Heinze S Herr C Gebauer R Schmidt-Weber CB Buters JTM An operational robotic pollen monitoring network based on automatic image recognition. Environ Res. 2020; 191: 110031. 10.1016/j.envres.2020.110031 32814105

[62] Sauvageat E Zeder Y Auderset K Calpini B Clot B Crouzy B Konzelmann T Lieberherr G Tummon F Vasilatou K Real-time pollen monitoring using digital holography. Atmos Meas Tech. 2020; 13: 1539–1550.

[63] Oteros J Sofiev M Smith M Clot B Damialis A Prank M Werchan M Wachter R Weber A Kutzora S Heinze S Herr CEW Menzel A Bergmann KC Traidl-Hoffmann C Schmidt-Weber CB Buters JTM Building an automatic pollen monitoring network (ePIN): Selection of optimal sites by clustering pollen stations. Sci Total Environ. 2019; 688: 1263–1274. 10.1016/j.scitotenv.2019.06.131 31726556

[64] Sofiev M Vira J Kouznetsov R Prank M Soares J Genikhovich E Construction of the SILAM Eulerian atmospheric dispersion model based on the advection algorithm of Michael Galperin. Geosci Model Dev. 2015; 8: 3497–3522.

[65] Scheifinger H Belmonte J Buters J Monitoring, Modelling and Forecasting of the Pollen Season. In: Sofiev M, Bergmann KC, (eds). Allergenic Pollen. Dordrecht: Springer; 2013. p. 71-126.

[66] Lo F Bitz CM Hess JJ Development of a Random Forest model for forecasting allergenic pollen in North America. Sci Total Environ. 2021; 773: 145590. 10.1016/j.scitotenv.2021.145590 33940736

[67] Zewdie GK Liu X Wu D Lary DJ Levetin E Applying machine learning to forecast daily Ambrosia pollen using environmental and NEXRAD parameters. Environ Monit Assess. 2019; 191 Suppl 2: 261. 10.1007/s10661-019-7428-x 31254085

[68] Muzalyova A Brunner JO Traidl-Hoffmann C Damialis A Forecasting Betula and Poaceae airborne pollen concentrations on a 3-hourly resolution in Augsburg, Germany: toward automatically generated, real-time predictions. Aerobiologia (Bologna). 2021; 37: 425–446.

[69] Cordero JM Rojo J Gutiérrez-Bustillo AM Narros A Borge R Predicting the Olea pollen concentration with a machine learning algorithm ensemble. Int J Biometeorol. 2021; 65: 541–554. 10.1007/s00484-020-02047-z 33188463

[70] Picornell A Hurtado S Antequera-Gómez ML Barba-González C Ruiz-Mata R de Gálvez-Montañez E Recio M Trigo MDM Aldana-Montes JF Navas-Delgado I A deep learning LSTM-based approach for forecasting annual pollen curves: Olea and Urticaceae pollen types as a case study. Comput Biol Med. 2024; 168: 107706. 10.1016/j.compbiomed.2023.107706 37989073

[71] Valipour Shokouhi B de Hoogh K Gehrig R Eeftens M Spatiotemporal modelling of airborne birch and grass pollen concentration across Switzerland: A comparison of statistical, machine learning and ensemble methods. Environ Res. 2024; 263: 119999. 10.1016/j.envres.2024.119999 39305973

[72] Plaza MP Oteros J Leier-Wirtz V Kolek F Menzel A Buters JTM Traidl-Hoffmann C Damialis A A multi-scale analysis of airborne Alternaria spore dispersal: influence of meteorology, land cover and air pollution. Agric For Meteorol. 2025; 372: 110716.

[73] Ratnayake MN Dyer AG Dorin A Tracking individual honeybees among wildflower clusters with computer vision-facilitated pollinator monitoring. PLOS ONE. 2021; 16: e0239504. 10.1371/journal.pone.0239504 PMC7877608 33571210

[74] Tofilski A Using geometric morphometrics and standard morphometry to discriminate three honeybee subspecies. Apidologie (Celle). 2008; 39: 558–563.

[75] Narcia-Macias CI Guardado J Rodriguez J IntelliBeeHive: An Automated Honey Bee, Pollen, and Varroa Destructor Monitoring System. In: 2024 International Conference on Machine Learning and Applications (ICMLA). IEEE; 2024: 845-850.

[76] O’Shea-Wheller TA Corbett A Osborne JL Recker M Kennedy PJ VespAI: a deep learning-based system for the detection of invasive hornets. Commun Biol. 2024; 7: 354. 10.1038/s42003-024-05979-z PMC10991484 38570722

[77] Bjerge K Mann HMR Høye TT Real‐time insect tracking and monitoring with computer vision and deep learning. Remote Sens Ecol Conserv. 2022; 8: 315–327.

[78] Shirali H Hübner J Both R Image-based recognition of parasitoid wasps using advanced neural networks. Giribet G, ed. Invertebr Syst. 2024; 38: IS24011. 10.1071/IS24011 38838190

[79] Spiesman BJ Gratton C Hatfield RG Hsu WH Jepsen S McCornack B Patel K Wang G Assessing the potential for deep learning and computer vision to identify bumble bee species from images. Sci Rep. 2021; 11: 7580. 10.1038/s41598-021-87210-1 PMC8027374 33828196

[80] Chen C Liu Z Pan Q Chen X Wang H Guo H Liu S Lu H Tian S Li R Shi W Genomic Analyses Reveal Demographic History and Temperate Adaptation of the Newly Discovered Honey Bee Subspecies Apis mellifera sinisxinyuan n. ssp. Mol Biol Evol. 2016; 33: 1337–1348. 10.1093/molbev/msw017 PMC4839221 26823447

[81] Dramburg S Marchante Fernández M Potapova E Matricardi PM The Potential of Clinical Decision Support Systems for Prevention, Diagnosis, and Monitoring of Allergic Diseases. Front Immunol. 2020; 11: 2116. 10.3389/fimmu.2020.02116 PMC7511544 33013892

[82] Papachristou P Söderholm M Pallon J Taloyan M Polesie S Paoli J Anderson CD Falk M A-297 - Unlocking Potential in Clinical Practice: A Comprehensive Assessment of an Artificial Intelligence Based Clinical Decision Support System for Cutaneous Melanoma Detection. EJC Skin Cancer. 2024; 2: 100111.

[83] Martinroche G Guemari A Apoil PA Allergen Chip Challenge: A nationwide open database supporting allergy prediction algorithms. J Allergy Clin Immunol. 2026; 157: 45–55. 10.1016/j.jaci.2025.09.017 41016485

[84] Matricardi PM Potapova E Forchert L Dramburg S Tripodi S Digital allergology: Towards a clinical decision support system for allergen immunotherapy. Pediatr Allergy Immunol. 2020; 31: 61–64. 10.1111/pai.13165 32017213

[85] Hassija V Chamola V Mahapatra A Singal A Goel D Huang K Scardapane S Spinelli I Mahmud M Hussain A Interpreting Black-Box Models: A Review on Explainable Artificial Intelligence. Cognit Comput. 2024; 16: 45–74.

[86] Kong E Cucco A Custovic A Fontanella S Machine learning in allergy research: A bibliometric review. Immunol Lett. 2026; 277: 107088. 10.1016/j.imlet.2025.107088 40930256

[87] Topaz M Peltonen LM Zhang Z Beyond human ears: navigating the uncharted risks of AI scribes in clinical practice. NPJ Digit Med. 2025; 8: 569. 10.1038/s41746-025-01895-6 PMC12460601 40993221

[88] Pham TC Luong CM Hoang VD Doucet A AI outperformed every dermatologist in dermoscopic melanoma diagnosis, using an optimized deep-CNN architecture with custom mini-batch logic and loss function. Sci Rep. 2021; 11: 17485. 10.1038/s41598-021-96707-8 PMC8410796 34471174

[89] Matricardi PM Monnati F Palmieri L Dramburg S Mesonjesi E Sfika I Villella V Arasi S Brighetti MA Potapova E Hoffmann TM Pelosi S Bregu B Lame B Charpin D Delgado L Fonseca JA Goksel O Kalpaklioglu F Nieto A Etiologic diagnosis of seasonal allergic rhinitis supported by artificial intelligence: The @IT-2020 project. J Allergy Clin Immunol. 2026; 158: 139–150. 10.1016/j.jaci.2026.03.011 41903764

[90] Matricardi PM Molecular allergology world atlas: Calling to bridge IgE microarrays and artificial intelligence. J Allergy Clin Immunol. 2026; 157: 73–76. 10.1016/j.jaci.2025.10.009 41167401

[91] Lammert J Dreyer T Mathes S Kuligin L Borm KJ Schatz UA Kiechle M Lörsch AM Jung J Lange S Pfarr N Durner A Schwamborn K Winter C Ferber D Kather JN Mogler C Illert AL Tschochohei M Expert-Guided Large Language Models for Clinical Decision Support in Precision Oncology. JCO Precis Oncol. 2024; 8: e2400478. 10.1200/PO-24-00478 39475661

[92] Holzmann C Karg J Reiger M Kharbal R Romano P Scheiwein S Khalfi C Muzalyova A Brunner JO Hammel G Damialis A Traidl-Hoffmann C Plaza MP Gilles S Clinical Benefits of a Randomized Allergy App Intervention in Grass Pollen Sufferers: A Controlled Trial. Allergy. 2025; 80: 1945–1955. 10.1111/all.16558 PMC12261868 40242867

[93] Matricardi PM Hoffmann T Dramburg S The “allergic nose as a pollen detector” concept: e-Diaries to predict pollen trends. Pediatr Allergy Immunol. 2023; 34: e13966. 10.1111/pai.13966 37366207

[94] Khoury P Oppenheimer J Bunyavanich S Ciaccio CE Portnoy J Preparing Allergists to Practice in 2050 Using Artificial Intelligence. J Allergy Clin Immunol Pract. 2025; 13: 2927–2935. 10.1016/j.jaip.2025.09.012 PMC12697010 40976355

[95] Young RA Martin CM Sturmberg JP Hall S Bazemore A Kakadiaris IA Lin S What Complexity Science Predicts About the Potential of Artificial Intelligence/Machine Learning to Improve Primary Care. J Am Board Fam Med. 2024; 37: 332–345. 10.3122/jabfm.2023.230219R1 38740483

[96] Hwang S Heo S Hong S Jung KH Cha WC Yoo J Multimodal AI-based 28-day mortality prediction of pneumonia patients at ED discharge: a multicenter study. Sci Rep. 2026; 16: 11761. 10.1038/s41598-026-42378-2 PMC13066529 41772120

[97] Razaghi M Hafez A Farina JM Scalia IG Pereyra M Abdelfattah FE Sheashaa H Awad K Lester SJ Ayoub C Arsanjani R Transforming clinical documentation with ambient artificial intelligence (AI) scribes: a narrative review of technology, impact, and implementation. Cardiovasc Diagn Ther. 2026; 16: 11. 10.21037/cdt-2025-454 PMC12973079 41815573

[98] Di Domizio J Girardin A Conrad C Seremet T Gilliet M Dermatology 2.0: Precision medicine for inflammatory skin diseases. J Eur Acad Dermatol Venereol. 2026; 40: 440–445. 10.1111/jdv.70251 PMC12933700 41453833

[99] Arasi S Castelli S Di Fraia M Villalta D Tripodi S Perna S Dramburg S Brighetti MA Conte M Martelli P Sfika I Travaglini A Verardo PL Villella V Matricardi PM @IT2020: An innovative algorithm for allergen immunotherapy prescription in seasonal allergic rhinitis. Clin Exp Allergy. 2021; 51: 821–828. 10.1111/cea.13867 33759270

[100] Liu TL Hetherington TC Stephens C McWilliams A Dharod A Carroll T Cleveland JA AI-Powered Clinical Documentation and Clinicians’ Electronic Health Record Experience: A Nonrandomized Clinical Trial. JAMA Netw Open. 2024; 7: e2432460. 10.1001/jamanetworkopen.2024.32460 PMC11380097 39240568

[101] Unlocking The Benefits of Clinical Decision Support Systems For Better Care. Ekagra Health. January 20, 2026. Accessed June 9, 2026. https://ekagrahealth.ai/benefits-of-clinical-decision-support-systems/.

[102] Truhn D Azizi S Zou J Cerda-Alberich L Mahmood F Kather JN Artificial intelligence agents in cancer research and oncology. Nat Rev Cancer. 2026; 26: 256–269. 10.1038/s41568-025-00900-0 41526721

[103] Matricardi PM Dramburg S Digital Allergology: From Theory to Practice. Cham: Springer Nature; 2025.

[104] Lenharo M ChatGPT turns two: how the AI chatbot has changed scientists’ lives. Nature. 2024; 636: 281–282. 10.1038/d41586-024-03940-y 39623037

[105] Iqbal U Tanweer A Rahmanti AR Greenfield D Lee LTJ Li YJ Impact of large language model (ChatGPT) in healthcare: an umbrella review and evidence synthesis. J Biomed Sci. 2025; 32: 45. 10.1186/s12929-025-01131-z PMC12057020 40335969

[106] Sulejmani P Negris O Aoki V Chu CY Eichenfield L Misery L Mosca A Orfali RL Aroman MS Stalder JF Trzeciak M Wollenberg A Lio P A large language model artificial intelligence for patient queries in atopic dermatitis. J Eur Acad Dermatol Venereol. 2024; 38: e531–e535. 10.1111/jdv.19737 38168874

[107] Ye F Zhang H Luo X Wu T Yang Q Shi Z Evaluating ChatGPT’s Performance in Answering Questions About Allergic Rhinitis and Chronic Rhinosinusitis. Otolaryngol Head Neck Surg. 2024; 171: 571–577. 10.1002/ohn.832 38796735

[108] Cherrez-Ojeda I Faytong-Haro M Alvarez-Muñoz P Larco JI de Arruda Chaves E Rojo I Moncayo CV Ramon GD Rodas-Valero G Kocatürk E Mosnaim GS Robles-Velasco K How accurate are ChatGPT-4 responses in chronic urticaria? A critical analysis with information quality metrics. World Allergy Organ J. 2025; 18: 101071. 10.1016/j.waojou.2025.101071 PMC12206104 40584686

[109] Abi-Rafeh J Toscano-Rivero D Mazer BD Can ChatGPT provide parent education for oral immunotherapy? Ann Allergy Asthma Immunol. 2025; 135: 87–90. 10.1016/j.anai.2025.04.011 40287013

[110] Høj S Thomsen SF Meteran H Sigsgaard T Meteran H Artificial intelligence and allergic rhinitis: does ChatGPT increase or impair the knowledge? J Public Health (Oxf). 2024; 46: 123–126. 10.1093/pubmed/fdad219 37968109

[111] Aggarwal A Tam CC Wu D Li X Qiao S Artificial Intelligence-Based Chatbots for Promoting Health Behavioral Changes: Systematic Review. J Med Internet Res. 2023; 25: e40789. 10.2196/40789 PMC10007007 36826990

[112] Peerbolte TF van Diggelen RJ van den Haak P Geurts K Evers LJ Bloem BR de Vries NM van den Berg SW Conversational Agents Supporting Self-Management in People With a Chronic Disease: Systematic Review. J Med Internet Res. 2025; 27: e72309. 10.2196/72309 PMC12421203 40857094

[113] Burkhart PV Sabaté E Adherence to long-term therapies: evidence for action. J Nurs Scholarsh. 2003; 35: 207. 14562485

[114] Tan LD Nguyen N Lopez E Peverini D Shedd M Alismail A Nguyen HB Artificial Intelligence in the Management of Asthma: A Review of a New Frontier in Patient Care. J Asthma Allergy. 2025; 18: 1179–1191. 10.2147/JAA.S535264 PMC12367921 40851769

[115] Jiang F Jiang Y Zhi H Dong Y Li H Ma S Wang Y Dong Q Shen H Wang Y Artificial intelligence in healthcare: past, present and future. Stroke Vasc Neurol. 2017; 2: 230–243. 10.1136/svn-2017-000101 PMC5829945 29507784

[116] Babel A Taneja R Mondello Malvestiti F Monaco A Donde S Artificial Intelligence Solutions to Increase Medication Adherence in Patients With Non-communicable Diseases. Front Digit Health. 2021; 3: 669869. 10.3389/fdgth.2021.669869 PMC8521858 34713142

[117] Health C for D and R. Artificial Intelligence-Enabled Medical Devices. FDA. Published online October 3, 2025. Accessed April 22, 2026.https://www.fda.gov/medical-devices/software-medical-device-samd/artificial-intelligence-enabled-medical-devices.

[118] Li Y Xiong Y Fan W Wang K Yu Q Si L van der Smagt P Tang J Chen N Sequential model for predicting patient adherence in subcutaneous immunotherapy for allergic rhinitis. Front Pharmacol. 2024; 15: 1371504. 10.3389/fphar.2024.1371504 PMC11294208 39101142

[119] Lv S Ye X Wang Z Xia W Qi Y Wang W Chen Y Cai X Qian X A randomized controlled trial of a mobile application-assisted nurse-led model used to improve treatment outcomes in children with asthma. J Adv Nurs. 2019; 75: 3058–3067. 10.1111/jan.14143 31241192

[120] Pena-Robichaux V Kvedar JC Watson AJ ext Messages as a Reminder Aid and Educational Tool in Adults and Adolescents with Atopic Dermatitis: A Pilot Study. Dermatology Research and Practice. 2010; 2010: 894258. 10.1155/2010/894258 PMC2946611 20885940

[121] Li L Wang Z Cui L Xu Y Lee H Guan K The efficacy of a novel smart watch on medicine adherence and symptom control of allergic rhinitis patients: Pilot study. World Allergy Organ J. 2023; 16: 100739. 10.1016/j.waojou.2022.100739 PMC9840975 36694622

[122] Do YH van Aalderen W Dellbrügger E Grenzbach C Grigg J Grittner U Haarman E Hernandez Toro CJ Karadag B Roßberg S Weichert TM Whitehouse A Pizzulli A Dramburg S Matricardi PM Objective detection of wheeze at home by parents through a digital device: usage patterns and relationship with SABA administration. Pediatr Pulmonol. 2025; 60: e27295. 10.1002/ppul.27295 PMC11715141 39451025

[123] Chan AHY Pleasants RA Dhand R Tilley SL Schworer SA Costello RW Merchant R Digital Inhalers for Asthma or Chronic Obstructive Pulmonary Disease: A Scientific Perspective. Pulm Ther. 2021; 7: 345–376. 10.1007/s41030-021-00167-4 PMC8589868 34379316

[124] Malaya E Piątkowska A Panek M Kuna P Kupczyk M Kardas G Medication adherence in allergic diseases and asthma: a literature review. Front Pharmacol. 2024; 15: 1488665. 10.3389/fphar.2024.1488665 PMC11646763 39687293

[125] Okata-Karigane U Hirota M Takahashi C Miyagawa A Ako R Nakajima S Futamura M Yonekura S Ogawa Y Inomata T Ishikawa T Ito Y Masaki K Sato S Katoh N Morita H Adachi T AI-Based Objective Severity Assessment of Atopic Dermatitis Using Patient Photos in a Real-World Setting: A Digital Biomarker Approach. Allergy. 2025; 80: 3185–3189. 10.1111/all.16586 PMC12590331 40390173

[126] Reis ZSN Pereira GMV Dias CDS Lage EM de Oliveira IJR Pagano AS Artificial intelligence-based tools for patient support to enhance medication adherence: a focused review. Front Digit Health. 2025; 7: 1523070. 10.3389/fdgth.2025.1523070 PMC12069381 40364851

